# Supplementary figures and images for: Meta-analysis of the efficacy and safety of Ginkgolide Meglumine Injection combined with Butylphthalide in the treatment of Acute Ischemic Stroke
Source: PLoS One. 2024 Jan 5;19(1):e0296508. doi: 10.1371/journal.pone.0296508 (PMC10769014; doi:10.1371/journal.pone.0296508)

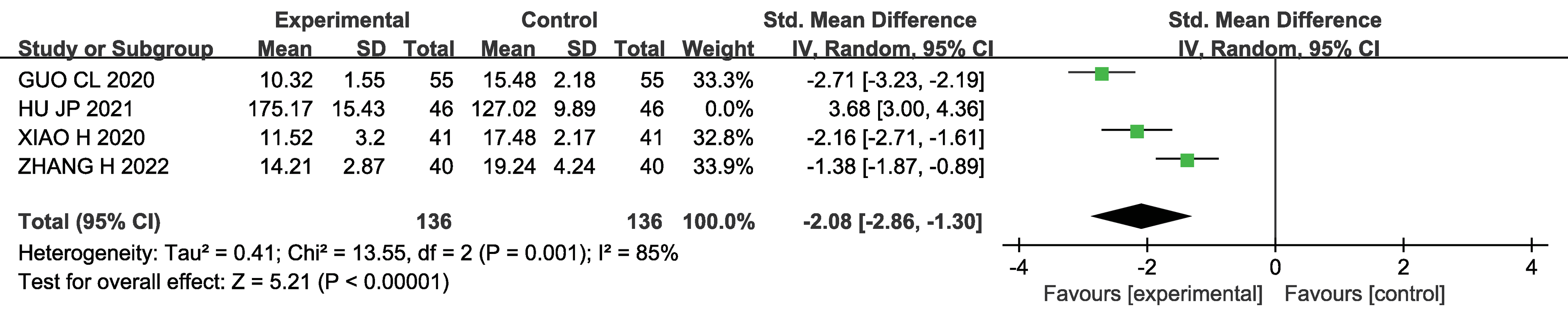

Supplement: S1 Fig — (TIF) [file pone.0296508.s002.tif]

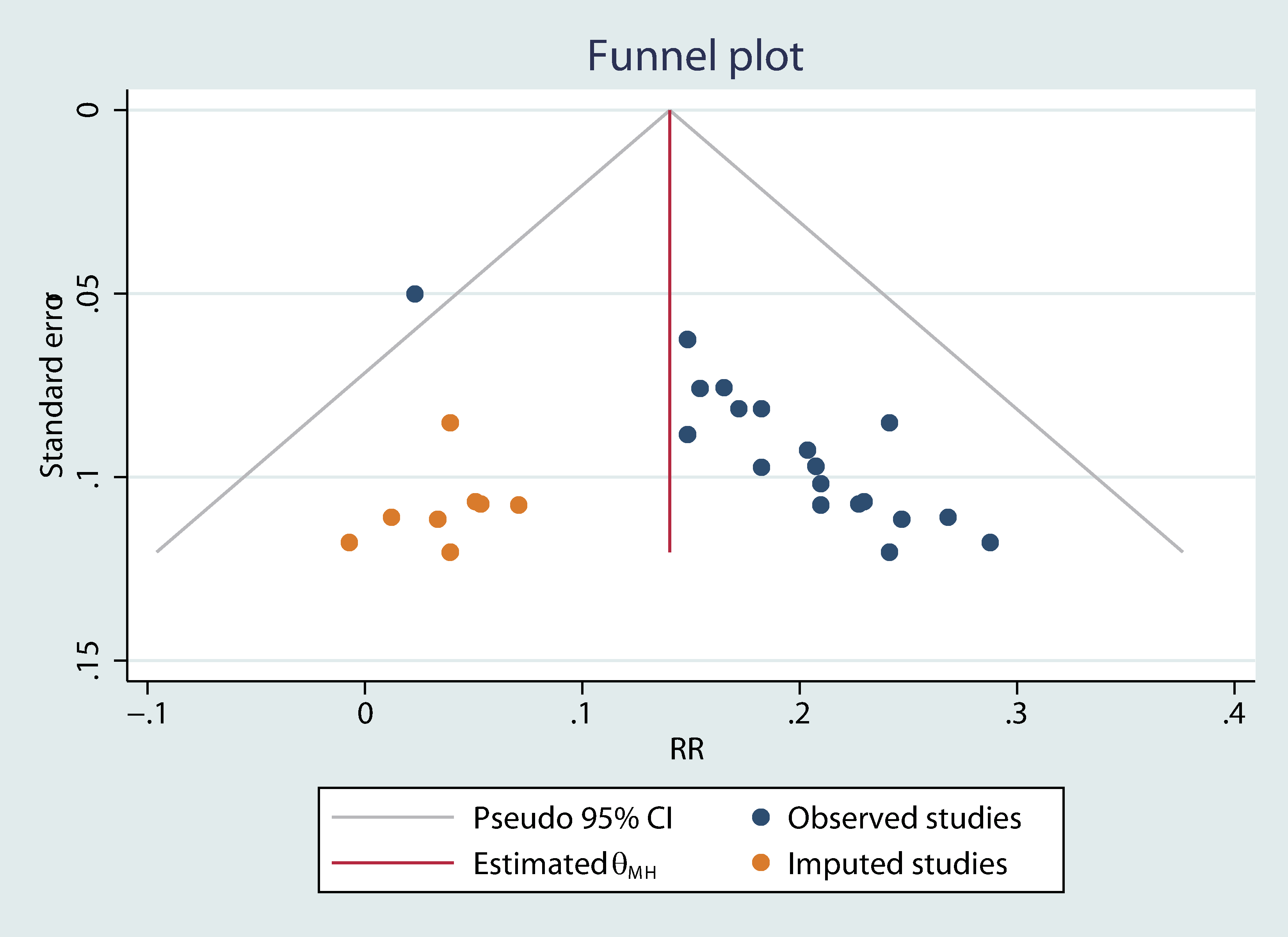

Supplement: S2 Fig — (TIF) [file pone.0296508.s003.tif]
