# Supplementary material for: Meta-analysis of the efficacy and safety of Ginkgolide Meglumine Injection combined with Butylphthalide in the treatment of Acute Ischemic Stroke
Source: PLoS One. 2024 Jan 5;19(1):e0296508. doi: 10.1371/journal.pone.0296508 (PMC10769014; doi:10.1371/journal.pone.0296508)
Supplement: S1 Table — (DOCX) [file pone.0296508.s004.docx]

**S1 Table. The detailed characteristics of Baseline Data.**

| **Study (years)** | **Group** | **Number** | **Men** | **Women** | **Age (years)**  **Mean ± sd** | | **Treatments** | **Outcome index** | **Time from onset to admission (mean ± sd)** | **Hypertension** | **Coronary disease** | **Atrial Fibrillation** | **Heart Failure** | **Diabetes** | **Hyperlipemia** | **Baseline NIHSS score** |  |
| --- | --- | --- | --- | --- | --- | --- | --- | --- | --- | --- | --- | --- | --- | --- | --- | --- | --- |
|  |  |  |  |  |  |  |  |  |  |  |  |  |  |  |  |  |  |
| CHEN ZZ  2021 | **E** | 44 | 23 | 21 | 52-74 | 63.21±2.32 | GMI (25mg, qd, 28d) + BSC (200 mg, tid, 28d) | 1. Traditional Chinese Medicine Syndrome Points 2. NIHSS score | 1-4 (2.53±0.37) | — | — | — | — | — | — | 16.53±2.76 |  |
|  | **C** | 44 | 22 | 22 | 53-76 | 64.15 ±2.41 | BSC (200mg, tid, 28d) |  | 1-4 (2.54±0.42) | — | — | — | — | — | — | 16.45±2.44 |  |
| FAN QQ  2018 | **E** | 43 | 24 | 19 | 41-77 | 55.3±9.7 | GMI (25mg, qd, 7-14d) + BSC (200mg, tid, 20) | 1. Effective rate 2. Hemodynamic indicators 3. Adverse reactions | 4.5-20.0 (13.6±2.5) | — | — | — | — | — | — | 14.4±5.2 |  |
|  | **C** | 43 | 25 | 18 | 42-78 | 56.1±9.4 | GMI (25mg, qd, 20) |  | 5.0-21.0 (14.0±2.6) | — | — | — | — | — | — | 14.8±6.7 |  |
| GUO CL  2020 | **E** | 55 | 26 | 29 | — | 56.12±5.17 | GMI (25mg, bid, 14d) + BI (25mg, bid, 14d) | 1. Effective rate 2. NIHSS/ ADLscore 3. NSE and Hcy levels 4. Adverse reactions | 17.81±2.94 | — | — | — | — | — | — | 8.91±2.21 |  |
|  | **C** | 55 | 29 | 26 | — | 57.17±5.41 | BI (25mg, bid, 14d) |  | 17.76±2.03 | — | — | — | — | — | — | 9.05±1.99 |  |
| GUO W  2022 | **E** | 42 | 22 | 20 | 39-74 | 58.32±10.83 | GMI (25mg, qd, 14d) + BI (25mg, qd, 14d) | 1. Effective rate 2. NIHSS score 3. Oxidative stress factors 3. Inflammatory factors 4. Adverse reactions | 1-6 (3.34±0.45) | 8 | — | — | — | 4 | — | 16.79±6.10 |  |
|  | **C** | 43 | 24 | 19 | 38-75 | 58.40±10.77 | BI (25mg, qd, 14d) |  | 1-6 (3.38±0.46) | 10 | — | — | — | 3 | — | 17.02±6.14 |  |
| HOU ZB  2020 | **E** | 60 | 31 | 29 | 48-81 | 65.37±10.69 | GMI (25mg, qd, 14d) + BI (25mg, bid, 14d) | 1. Effective rate 2. NIHSS score 3. Adverse reactions | 2.5-12 (6.0±0.57) | 42 | — | — | — | 20 | 6 | 13.28±4.55 |  |
|  | **C** | 60 | 32 | 28 | 49-80 | 65.08±10.41 | BI (25mg, bid, 14d) |  | 2.4-13 (6.2±0.43) | 48 | — | — | — | 23 | 5 | 13.06±4.79 |  |
| HU JP  2021 | **E** | 46 | 35 | 11 | — | 62.27±3.68 | GMI (25mg, qd, 28d) + BSC (60mg, bid, 28d) | 1. Effective rate 2. NIHSS/Fugl-Meyer score、Barthel index 3. GABA/Gly/Hcy levels 4. Content of neurotransmitters and cytokines | 4.12±1.22 | 20 | — | — | — | 12 | 9 | 14.53±3.97 |  |
|  | **C** | 46 | 37 | 9 | — | 62.35±3.59 | BSC (60mg, bid, 28d) |  | 4.23±1.06 | 22 | — | — | — | 13 | 8 | 14.35±3.69 |  |
| JIAO J  2023 | **E** | 30 | — | — | 57-67 | 61.3±2.1 | GMI (25mg, qd, 14d) + BI (25mg, bid, 14d) | 1. Effective rate 2. NIHSS/quality of life score 3. Inflammatory factors | 1.15±0.22 | — | — | — | — | — | — | — |  |
|  | **C** | 30 | — | — | 58-68 | 61.1±1.8 | BI (25mg, bid, 14d) |  | 1.13±0.18 | — | — | — | — | — | — | — |  |
| LIANG CC 2022 | **E** | 35 | 20 | 15 | 45-92 | 63.91±1.23 | GMI (25mg, qd, 14d) + BI (25mg, bid, 14d) | 1. Effective rate 2. NIHSS score 3. Hemorheology index 4. Adverse reactions | 5.43±1.32 | — | — | — | — | — | — | 25.98±2.23 |  |
|  | **C** | 35 | 19 | 16 | 45-92 | 63.92±1.21 | BI (25mg, bid, 14d) |  | 5.44±1.31 | — | — | — | — | — | — | 26.32±2.17 |  |
| LI NJ  2020 | **E** | 51 | 28 | 23 | 31-78 | 56.32±6.32 | GMI (30mg, bid, 7d) + BI (25mg, bid, 7d) | 1. Effective rate 2. NIHSS、BI score 3. BNP and D-dimer levels 4. Adverse reactions | <24 | 34 | 7 | — | — | 14 | — | 15.6±2.2 |  |
|  | **C** | 51 | 29 | 22 | 32-79 | 56.40±6.35 | BI (25mg, bid, 7d) |  | <24 | 35 | 6 | — | — | 13 | — | 15.5±2.3 |  |
| LIU BC  2021 | **E** | 40 | 20 | 20 | 40-68 | 53.21±1.58 | GMI (25mg, qd, 14d) + BI (25mg, bid, 60d) | 1. Effective rate 2. ALD/ESS score 3. Adverse reactions | 5.52±1.62 | 14 | 11 | — | — | — | 15 | — |  |
|  | **C** | 40 | 21 | 19 | 40-69 | 53.19±1.62 | BI (25mg, bid, 60d) |  | 5.48±1.59 | 14 | 12 | — | — | — | 14 | — |  |
| LIU J  2019 | **E** | 34 | 19 | 15 | 46-73 | 57.9±4.3 | GMI (25mg, bid, 14d) + BI (25mg, bid, 14d) | 1. Effective rate 2. NIHSS score 3. Adverse reactions | — | — | — | — | — | — | — | 9.30±2.1 |  |
|  | **C** | 34 | 16 | 18 | 43-75 | 56.3±5.4 | BI (25mg, bid, 14d) |  | — | — | — | — | — | — | — | 8.78±3.0 |  |
| LIU Y  2021 | **E** | 30 | 16 | 14 | 49-87 | 68.3±9.7 | GMI (25mg, qd, 14d) + BSC (25mg, bid,14d) | 1. NIHSS/mRS score | — | — | — | — | — | — | — | 5.13±2.047 |  |
|  | **C** | 30 | 16 | 14 | 48-85 | 66.4±11.5 | GMI (25mg, qd, 14d) |  | — | — | — | — | — | — | — | 4.97±1.829 |  |
| LI X  2021 | **E** | 36 | 22 | 14 | — | 50.5±4.7 | GMI (25mg, qd, 14d) + BI (25mg, bid, 14d) | 1. Effective rate 2. NIHSS score | 13.94±5.25 | — | — | — | — | — | — | 20.4±4.5 |  |
|  | **C** | 36 | 20 | 16 | — | 50.3±4.9 | BI (25mg, bid,14d) |  | 13.92±5.23 | — | — | — | — | — | — | 20.1±4.8 |  |
| LUO W  2022 | **E** | 64 | 35 | 29 | 48-76 | 64.3±7.9 | GMI (25mg, qd, 14d) + BSC (200mg, tid, 20d) | 1. Effective rate 2. NIHSS/ADL score 3. Inflammatory factors 4. Adverse reactions | ≤4 | — | — | — | — | 23 | 19 | 18.3±4.9 |  |
|  | **C** | 63 | 33 | 30 | 49-78 | 64.5±8.7 | BSC (200mg, tid, 20d) |  | ≤4 | — | — | — | — | 24 | 18 | 18.5±3.7 |  |
| QIN RF  2020 | **E** | 60 | 32 | 28 | 48-82 | 67.39±2.53 | GMI (25mg, qd, 14d) + BI (25mg, bid, 14d) | 1. Effective rate 2. NIHSSscore | 7-30 (19.35±3.67) | — | — | — | — | — | — | 17.48±2.75 |  |
|  | **C** | 60 | 31 | 29 | 49-81 | 67.75±2.35 | BI (25mg, bid, 14d) |  | 8-29 (19.65±3.13) | — | — | — | — | — | — | 17.37±2.53 |  |
| QIU XL  2022 | **E** | 53 | 27 | 26 | 46-80 | 63.93±2.25 | GMI (25mg, qd, 14d) + BI (25mgl, bid, 14d) | 1. Effective rate 2. NIHSS/ESS/MMSE/ADL score 3. Clinical indicators 4. Adverse reactions | 2-8 (5.55±0.13) | — | — | — | — | — | — | 10.66±1.37 |  |
|  | **C** | 53 | 30 | 23 | 45-78 | 63.47±2.19 | BI (25mg, bid, 14d) |  | 3-8 (5.23±0.12) | — | — | — | — | — | — | 10.59±1.36 |  |
| SHI XJ  2021 | **E** | 63 | 32 | 31 | 45-81 | 65.13±7.29 | GMI (25mg, qd, 14d) + BI (25mg, bid, 14d) | 1. Effective rate 2. NIHSS/BI score 3. Hemorheology indicators 4. Number of lateral Cyclic number 5. Classification of collateral circulation | <48 | — | — | — | — | — | — | 13.36±2.58 |  |
|  | **C** | 63 | 35 | 28 | 45-79 | 64.23±6.18 | BI (25mg, bid, 14d) |  | <48 | — | — | — | — | — | — | 12.87±1.46 |  |
| SONG CY 2022 | **E** | 60 | 32 | 28 | 46-62 | 53.69±2.83 | GMI (25mg, qd, 14d) + BSC (200mg, tid, 14d) | 1. NIHSS score 2. Oxidative stress factors 3. Inflammatory factors | 3-16 (9.52±1.15) | — | — | — | — | — | — | 10.61±2.69 |  |
|  | **C** | 60 | 35 | 25 | 45-62 | 53.56±2.81 | BSC (200mg, tid, 14d) |  | 2-16 (9.31±1.12) | — | — | — | — | — | — | 10.78±2.81 |  |
| SONG YQ 2021 | **E** | 52 | 30 | 22 | 53-78 | 63.55±3.21 | GMI (25mg, qd, 14d) + BI (25mg, bid, 14d) | 1. Effective rate 2. NIHSS score 3. Hemorheology index 4. Adverse reactions | 9-22 (14.65±2.11) | — | 20 | 22 | 10 | — | — | 17.65±2.14 |  |
|  | **C** | 52 | 31 | 21 | 55-78 | 63.52±3.18 | BI (25mg, bid, 14d) |  | 9-22 (14.62±2.07) | — | 18 | 23 | 11 | — | — | 17.68±2.17 |  |
| WENG GM 2019 | **E** | 30 | 17 | 13 | 42-75 | 63.55±11.82 | GMI (25mg, qd, 90d) + BSC (200mg, tid, 90d) | 1. Effective rate 2. NIHSS score 3. Adverse reactions | 4-39 (18.58±6.03) | 8 | — | — | — | 6 | — | 10.55±4.28 |  |
|  | **C** | 30 | 18 | 12 | 43-78 | 63.49±11.92 | BSC(200mg, tid, 90d) |  | 4-48 (18.39±5.98) | 9 | — | — | — | 7 | — | 10.62±4.37 |  |
| XIAO H  2020 | **E** | 41 | 28 | 13 | 42-85 | 62.15±6.45 | GMI (25mg, qd, 14d) + BI (25mg, bid, 14d) | 1. Effective rate 2. NIHSS score 3. Coagulation function 4. hcy and D-dimer levels 5. Prognosis | 6~72 | 9 | — | — | — | 9 | 6 | 16.15±3.55 |  |
|  | **C** | 41 | 29 | 12 | 39-80 | 61.37±6.44 | BI (25mg, bid, 14d) |  | 6~72 | 9 | — | — | — | 7 | 5 | 15.33±3.52 |  |
| YANG ZM 2020 | **E** | 48 | 25 | 23 | 20-76 | 58.65±2.16 | GMI (25mg, qd, 60d) + BI (25mg, bid, 60d) | 1. Effective rate 2. NIHSS/ADL score 3. Adverse reactions | 2-12 (5.03±0.65) | 11 | 4 | — | — | — | 7 | 14.95±1.16 |  |
|  | **C** | 48 | 22 | 26 | 20-76 | 58.61±2.13 | BI (25mg, bid, 60d) |  | 2-12 (5.01±0.63) | 12 | 4 | — | — | — | 7 | 14.92±1.13 |  |
| ZHANG H 2022 | **E** | 40 | 28 | 12 | — | 58.42±5.41 | GMI (25mg, qd, 14d) + BSC (200mg, tid, 14d) | 1. Effective rate 2. NIHSS/ADL/MMSE score 3. Blood monitoring indicators 4. Adverse reactions | 41.62±1.43 | — | — | — | — | — | — | 28.73±3.16 |  |
|  | **C** | 40 | 26 | 14 | — | 57.15±7.21 | BSC (200mg, tid, 14d) |  | 42.43±1.52 | — | — | — | — | — | — | 29.24±3.21 |  |
| ZHANG Y 2022 | **E** | 49 | 27 | 22 | 46-75 | 62.7±5.5 | GMI (25mg, qd, 7-14d) + BSC (200mg, tid, 20d) | 1. NIHSS score 2. Hemodynamic related indicators 3. Inflammatory factors 4. Adverse reactions 5. Late ecurernce | ≤24 | 30 | — | 5 | — | 18 | — | 19.88±1.425 |  |
|  | **C** | 49 | 26 | 23 | 47-73 | 61.3±5.2 | BSC (200mg, tid, 20d) |  | ≤24 | 32 | — | 6 | — | 17 | — | 19.67±1.35 |  |
| ZHENG WW 2018 | **E** | 45 | 23 | 22 | — | 57.43±7.29 | GMI (25mg, bid, 14d) + BI (25mg, bid, 60d) | 1. Effective rate 2. ADL/ESS score 3. Adverse reactions | 5.68±2.29 | 12 | 5 | — | — | — | 8 | — |  |
|  | **C** | 45 | 24 | 21 | — | 57.31±7.21 | BI (25mg, bid, 60d) |  | 5.59±2.17 | 10 | 6 | — | — | — | 10 | — |  |

**Explanation of Acronyms:** E: experimental group; C: control group; GMI: Ginkgolide Meglumine Injection; BI: Butylphthalide Injection; BSC: Butylphthalide Soft Capsules; NIHSS score: National Institutes of Health Stroke Scale score, ranging from 0 to 42 points, with higher scores indicating more severe neurological damage; ADL score: Activities of Daily Living score, ranging from 0 to 100 points, with higher scores indicating better daily living abilities; CRP: C-Reactive Protein, a non-specific inflammatory marker; TNF-α: tumor necrosis factor-alpha; NSE: neuron-specific enolase; mRS score: the modified Rankin score scale, used to evaluate the neurological recovery status of stroke patients, divided into seven levels, with lower scores indicating better neurological function; ESS score: Epworth sleepiness scale; MMSE score: Mini-mental State Examination; BNP: Brain natriuretic peptide.
