# Supplementary material for: Meta-analysis of the efficacy and safety of Ginkgolide Meglumine Injection combined with Butylphthalide in the treatment of Acute Ischemic Stroke
Source: PLoS One. 2024 Jan 5;19(1):e0296508. doi: 10.1371/journal.pone.0296508 (PMC10769014; doi:10.1371/journal.pone.0296508)
Supplement: S1 File — (DOCX) [file pone.0296508.s005.docx]

**Identification of new studies via other methods**

**Previous studies**

**Identification of new studies via databases and registers**

Studies included in previous version of review (n = 0)

Reports of studies included in previous version of review (n = 0)

Records identified from*:

Databases (n = 69)

Registers (n = 0)

Records removed *before screening*:

Duplicate records removed (n = 37)

Records marked as ineligible by automation tools (n = 3)

Records removed for other reasons (n = 0)

Records identified from:

Websites (n = 0)

Organisations (n = 0)

Citation searching (n = 0)

etc.

**Identification**

Total studies included in review

(n = 25)

Reports of total included studies

(n = 0)

Reports assessed for eligibility

(n = 0)

Reports sought for retrieval

(n = 0)

Records screened

(n = 28)

Records excluded**

(n = 3)

Reports not retrieved

(n = 0)

Reports sought for retrieval

(n = 0)

Reports not retrieved

(n = 0)

**Screening**

Reports excluded:

Reason 1 (n = 0)

Reason 2 (n = 0)

Reason 3 (n = 0)

etc.

Reports excluded:

Reason 1 (n = 0)

Reason 2 (n = 0)

Reason 3 (n = 0)

etc.

Reports assessed for eligibility

(n = 0)

New studies included in review

(n = 25)

Reports of new included studies

(n = 0)

**Included**

*Consider, if feasible to do so, reporting the number of records identified from each database or register searched (rather than the total number across all databases/registers).

**If automation tools were used, indicate how many records were excluded by a human and how many were excluded by automation tools.

From: Page MJ, McKenzie JE, Bossuyt PM, Boutron I, Hoffmann TC, Mulrow CD, et al. The PRISMA 2020 statement: an updated guideline for reporting systematic reviews. BMJ 2021;372:n71. doi: 10.1136/bmj.n71. For more information, visit: <http://www.prisma-statement.org/>
